# Medicine and Psychology
1Address delivered at at meeting of the Bristol Medico-Chirurgical Society, January 11th, 1922.


**Published:** 1922-06

**Authors:** John Herbert Parsons

**Affiliations:** Surgeon, Royal London Ophthalmic Hospital; Ophthalmic Surgeon, University College Hospital


*" Scire est nescire, nisi id me*
*Scire alius sciret.*

MARCH AND JUNE, 1922.

### MEDICINE AND PSYCHOLOGY.[1]

BY

SIR JOHN HERBERT PARSONS, C.B.E., F.R.C.S., F.R.S.,
*Surgeon, Royal London Ophthalmic Hospital; Ophthalmic Surgeon, University College Hospital.*

" *Psychologus nemo nisi physiologus.*"—JOHANNES MÜLLER.

THE scientific study of the behaviour of human beings has been neglected by the medical profession. Behaviour is the end result and outward manifestation of complex mental processes. Only a small proportion of medical men have paid much attention to the laws which govern mental processes, and these few have been principally alienists, devoted to the study of grossly pathological mental states. Medical students are compelled to spend years in acquiring a knowledge of the structure and functions of the human body, but are left with little or no instruction in the activities of the mind

[1] Address delivered at a meeting of the Bristol Medico-Chirurgical Society, January 11th, 1922.

which the body subserves. No one would dream of teaching pathology without a preliminary course of physiology, yet every doctor is daily called upon to treat neuroses and psychoses with quite inadequate training. The stress of modern life, culminating in the War, has greatly increased the amount of mental instability, more particularly in those less severe forms which come under the observation of general practitioners and others who are least well equipped to deal with them.

There has thus been created an imperative demand for instruction in psycho-pathology. The demand has coincided with the astonishing development of a new school of psychology, founded by Professor Freud, of Vienna. The attractiveness for various reasons of Freud's theories and speculations has led to a flood of literature on the subject, much of it written by facile authors ignorant of the well-established principles of the older psychology. Freudism has been elevated into a cult, its truths have been overlaid with speculations, and the unconscious has usurped an autonomy which it is scarcely likely to maintain.

If the "new psychology" provides an incomplete and unsatisfactory basis for the psychological training of the medical man it becomes necessary to define the psychology which fills the rôle adequately. In order to do so it will be necessary, in as few words as possible, to explain what I regard as the scope of psychology *as a science*, for the term is used in such a haphazard manner that much confusion results.

*The Scope of Psychology.*—Psychology is the offspring of philosophy, and it is by almost imperceptible stages that it has germinated and finally attained an independent existence. Philosophy is the manifestation of the irresistible impulse of human beings to account for themselves, the universe and their place in it. The evolution of free ideas and conceptual thought has endowed humanity with imagination and an immense capacity for devising speculations.

The gradual emergence of science from philosophy is the greatest achievement of human thought. It has resulted from a gradually increasing control of the flights of speculative imagination. Only in this respect do the fundamental principles of science differ from those of philosophy. For science, like philosophy, is built upon a foundation of speculation, and the all-important question is, What is the difference between philosophical and scientific speculation? It is convenient to distinguish between them verbally, and I propose to call the former speculation and the latter hypothesis. I think that the essential difference between speculation and hypothesis is utility. Utility is, and always has been, the prime motive force of living organisms and the predominant condition of evolution and progress. There is good biological evidence to support this view, for from the amœba upwards success in action is attained through the principle of "trial and error." Now, scientific hypothesis is the application on the highest plane of the principle of "trial and error," and an hypothesis is of no scientific value—and, therefore, is not an hypothesis in my restricted use of the word—unless it leads to success, *i.e.*, verifies established relations ("facts") and can be projected into the establishment of hitherto undiscovered relations.

Increased precision of thought, brought about chiefly by advance in knowledge of astronomy, mathematics and physics, together with the more slowly advancing knowledge of biology and physiology, due chiefly to physicians, led to the replacement in the domain of science of vague and varied speculations about the soul by the concept of the mind. Mind has had almost as varied connotations as soul, but in the past it has generally been regarded as equivalent to consciousness, so that psychology has been defined as the science of the mind or the science of conscious processes. Galen first showed that the brain was intimately concerned with conscious processes, and indeed taught that it is the seat of the " soul " and the medium through which sensations are produced. This was a great step in advance. It established a scientific relationship between the body and conscious processes, and so initiated a scientific psychology founded upon physiology.

Mind, however, is too general a term to apply as a satisfactory criterion of the meaning of psychology. Mind includes all conscious processes, and without further limitation would embrace all knowledge and all the sciences, for none is possible without the activity of the mind.

Consciousness, on the other hand, is too narrow, for psychology deals with the whole mental history of the individual, and consciousness includes only such mental processes as rise above the threshold, the waves on the surface of the stream. At a time when the Unconscious plays so large a part in modern psychology to labour this point it is scarcely necessary. I need only remind you that subliminal mental processes occur temporally in sleep, in inattention, in oblivescence, and spatially in the fringe of the field of consciousness ; that the unconscious includes what James Ward has called sub-presentations ; that it is the seat of inherited instincts and acquired habits ; and that it is a reservoir of suppressed ideas in the form of memory images, ideas which are inhibited in the interests of the integration of thought, just as Sherrington has taught us that reflexes are integrated by inhibitory processes, and the great mass of repressed complexes upon which Freud and his followers lay so much stress. We have learnt to recognise that the stream of mental processes is continuous, rising rhythmically or at intervals above the threshold of consciousness. Mind is subjectively continuous ; only as the stimuli of interest, meaning, or suggestion succeed in forcing presentational, conative or ideational objects into the focus of attention do mental processes appear to be objectively discontinuous. [1]

The objections to the term "behaviour" as a criterion of psychology, apart from the unimportant fact that the term is commonly applied to non-living as well as to living things, has the great disadvantage that it lays stress upon the objective aspect of mental processes. Behaviour has hitherto always implied the behaviour of other animals or other people, and one's own behaviour implicitly indicates our activities as they appear to other people; and although it is not only legitimate, but very desirable, to define accurately the scientific connotation of words adopted from common parlance, as McDougall has well insisted, yet too great a dislocation of the generally accepted significance is undesirable, especially in a fundamental definition.

Nearly all psychologists agree that pure psychology is fundamentally concerned with the subjective aspect of mental processes. And it can scarcely be otherwise; for it is impossible to include the objective aspects as an integral part of the science without invading domains best left under the jurisdiction of other sciences, *e.g.* natural history, biology, etc. At first sight this appears to limit psychology to the old introspective psychology, for all will admit that the individual is conscious only of his own consciousness. And since the most perfect knowledge of a single individual's mental processes does not constitute a science, it would appear that a science of psychology is impossible. But while the objective aspects of mental processes in others should not constitute an integral part of pure

[1] We regret that we are obliged to omit the critical review of definitions of psychology by F. Ward, G. F. Stout and others.

psychology, it must be admitted—at worst as a mere hypothesis, if need be—that these objective processes are legitimate data for the elaboration of the science. This too can be hardly otherwise ; for it is clear that even in introspection the mental processes analysed *ipso facto* become objects. It is unavoidable that they thereby become changed in character, and it may well be doubted whether the data which, thus changed, they afford are more reliable than the data derived by judicious inference from the " behaviour " of other individuals.

It suffices for practical purposes if definition indicates the general scope of the science and its limitations in such simple phraseology as to be readily understood and as free as possible from ambiguity. For here again utility is the criterion, and definitions are for the uninitiated.

It seems to me that, provisionally, most of the objections urged against previous definitions would be avoided by the following : *Psychology is the science which treats of the laws of the evolution, organization, and manifestations of the mental processes occurring in living organisms in their relation to the organisms in which they occur.*

*The Data of Psychology.*—In defining psychology great stress has been laid upon the necessity for limiting its scope to the *subjective* aspects of mental processes. In dealing with the data of psychology it is important to realize that *all* the data are psychologically *objective*, and that the laws of psychology are merely inferences from observed relations (" facts ").

In no science is it more important than in psychology rests should conform to the best authenticated principles of those other sciences which are less open to criticism. Like other sciences it must proceed from the known to the unknown.

Now, the fundamental known fact for psychology as a science is that the only immediate knowledge of mental processes which I as an individual possess is that they are inherent in me *as a living organism.*

The study of the physiology of the central nervous system and organs of special sense has produced a vast volume of evidence as to the origin of sensations and the functions of the various parts of the nervous system in their co-ordination and integration. Research has elucidated many of the chief functions of the spinal cord, the basal ganglia, and the cortex cerebri, and though our knowledge is still very imperfect, all neurologists agree on certain fundamental points. Among these is the deduction that in man the cortex cerebri is the chief seat of those nervous processes which, in some manner as yet unknown, give rise to conscious mental processes. It is further agreed that motor activities are the responses of the central nervous system to the sensory impulses which impinge upon it : that both sensory impulses and motor responses are co-ordinated and integrated in highly complex ways, resulting in synergic activities of the organism which form at any rate a very large part of what is called its behaviour. Further, it is known that these activities of the nervous system are profoundly modified and influenced by the action of glandular secretions. It is a legitimate scientific hypothesis, founded upon these facts, that both conscious processes and behaviour are contingent upon nervous processes. Biologists and physiologists indeed accept this as their working hypothesis. It is only when the higher mental processes—ideation, conceptual thought, imagination, and so on—are the subject of inquiry that this hypothesis is tacitly ignored or denied and the philosopher steps in with his esoteric speculations. It seems easy to admit that sensations give rise to perceptions, though the physiologist shirks the psychological element of *meaning*

involved in the latter, but it is difficult to conceive of the neurological basis of a flight of imagination. Yet if psychology is a science it must proceed from the known to the unknown, and until knowledge has advanced to that stage at which the unknown becomes amenable to the resources of scientific investigation it is not a fit subject for scientific treatment.

If then we accept as a fundamental hypothesis that mental processes are correlated with nervous processes the biological evolution of the nervous system in various species becomes important to psychology; and further, if nervous processes and the mental processes to which they give rise lead to characteristic motor activities and behaviour, these become available as evidence of the mental processes of other individuals and of animals. The field of psychological investigation is thus widely extended, embracing (1) animals (*comparative psychology*), especially from the evolutionary point of view (*phylogenetic psychology*); (2) human individuals, in the objective sense of *other* individuals, including the development of human minds (*ontogenetic psychology*); (3) groups of individuals—for the behaviour of individuals acting together in groups shows specific peculiarities (*collective psychology*); (4) human races, primitive and advanced, including their cultural development (*anthropological psychology*); (5) the effects of disease upon mental processes, including neuroses, psychoses, criminology, etc. (*psycho-pathology*).

Finally, we arrive at what was historically primary, though it more or less unconsciously utilized much of the evidence derived from the sources mentioned, viz. *introspective psychology*, which must necessarily be the final court of appeal: (1) because it is the unique source of our knowledge of what consciousness is; (2) because through it alone can we directly investigate and analyse conscious processes and our own activities and behaviour as the manifestations of our own mental processes, thus obtaining a criterion in the behaviour of others for their mental processes. As Stout says of the validity of this criterion : "All depends on accurate resolution of his own complex consciousness into its constituents, and on re-compounding these in such a way and in such proportions as to explain the nature and order of the signs which indicate to him the mental processes of others."[1]

The mental processes in various individuals vary owing to acquired characteristics derived from tradition, social environment, and individual life-history, *i.e.* the previous experience of the individual.

The study of comparative psychology, collective psychology, anthropological psychology, and psycho-pathology has led of recent years to an appreciation of the predominant importance of the instincts as the prime motive forces of human as well as of animal behaviour. The application of the theory of evolution to the mind, begun by Darwin himself, is chiefly responsible for this great advance. Only by the study of behaviour in animals, savages, groups of men and women, and the subjects of neuroses and psychoses, was it possible to attain any sure knowledge of the instincts and their potency : for these are least of all submissible to the method of introspective analysis. Phylogenetically primitive mental states, such as the instincts, tend to be suppressed from consciousness and relegated to the unconscious. Only when they are excited by appropriate perceptive stimuli do they issue in impulsive conative acts, accompanied by conscious affective tone. The primitive emotions which colour instinctive reactions are inchoate and defy precise analysis by intro-spection. Nevertheless, introspection is the final and crucial

[1] *Analytic Psychology*, vol. i., p. 15.

test of the psychological significance of mental states from the subjective point of view. Guided by the indications derived from behaviour in animals, and so on, it conclusively supports the view of the predominant importance of the instincts as springs of conduct in men.

Psycho-pathology has carried the advance still further forwards. It has shown that the commonest forms of mental disorder—hysteria, neuroses, etc.—are due to conflicts in which the instincts war one with the other or with the canons of conventional behaviour. Owing to the painful nature of the conflict it is suppressed from consciousness and lies hidden in the unconscious. It is none the less potent in influencing behaviour, which thus becomes disordered. It cannot be denied that psychology owes an immense debt to Freud for the fundamental principles of his theory. Few will deny the general truth of his views on the suppression of conflicts, on sublimation, etc., and on the principle which lies at the root of psycho-analysis. They are founded on a secure scientific basis. It is in their elaboration that he and his followers quit science and launch out into speculation of more than doubtful validity.

Freud's theory is an example of the method of explaining the infinite intricacies of human behaviour by an appeal to a single principle. Hobbes found the key to the riddle of human nature in fear; Bentham, the Mills, and the other Utilitarians in the pursuit of happiness; Comte and the Positivists in altruism. Others have found the all-embracing principle in some special characteristic of the herd instinct, stress being variously laid upon imitation (Bagehot, Tarde, le Bon, E. A. Ross, and others), sympathy or suggestion, the last mentioned being specially applied to psycho-pathology by the old and new Nancy schools. There can be no doubt that this artificial simplification of the highly complex problem has been useful in stimulating thought and investigation and in eliciting valuable results. There can be equally no doubt that it leads to paradoxes which prove the fundamental unreliability of the method. Freud's " sexual " man will prove in the long run to be as impotent as those other theoretical monstrosities, the " economic " and the " ethical " man.

If this sketch of the scope and data of psychology is correct, it is clear that the science of psychology must be founded upon biology and physiology, and the teachers of psychology should be selected from men who have had a thorough training in these subjects. Yet when we consider the teachers of psychology in our Universities to-day we find that this is the exception rather than the rule. Most of them are the products of a mediæval classical education, and have devoted their lives to the study of philosophy rather than science. Further, just as general pathology is one of the main sources of knowledge of general physiology, so psycho-pathology is one of the main sources of our knowledge of psychology. Hence it is eminently desirable that teachers of psychology should also be fully qualified medical men. Indeed, there is no other class of men so well equipped by training from which to select specialists in psychology. Some of our Universities are at last recognising this fact. On the other hand, it is undesirable that psycho-pathologists who have not devoted exhaustive attention to general psychology should be regarded as the authoritative exponents of the science. Their purview is too narrow and their perspective is warped.

For my own part I should like to see psychology ruthlessly divorced from metaphysics and philosophy, and given a status of its own in the Faculty of Science. The curriculum in psychology, in addition to a preliminary grounding in general science, should contain exhaustive courses of biology and physiology, and only those who have passed an intermediate examination in these subjects should be allowed to present themselves for examination in psychology. The University of Bristol is pre-eminently fitted to establish such a reform, for its late Professor of Psychology, Professor C. Lloyd Morgan, is, without exception, the greatest living biological psychologist. Such an example would make it impossible for psychology to be taught in any British University by those who have not had an efficient training in biology and physiology.

Further, I am strongly of opinion that every medical student should be instructed in the rudiments of psychology, not only because he will find it useful in his professional career, but also because the scientific principles of psychology which he will thus acquire will increase his interest in the behaviour of his fellow-creatures and enable him to propagate the gospel to those among whom he lives. He will cease to be gulled by the false so-called psychology which is rampant, and will exercise a steadying influence upon the minds of those with whom he comes in contact. I think that this elementary course of psychology should follow upon his physiology course. Towards the end of the curriculum he should have a course of instruction in psycho-pathology.

It will doubtless be objected that the medical student's curriculum is already overcrowded. It is impossible to discuss this thorny question now. I will merely state that in my opinion a thorough revision of the whole medical curriculum is inevitable in the not distant future. Such a revision will prune the qualifying curriculum and examinations of many useless smatterings of knowledge, leaving time for subjects of greater practical importance. It is no longer possible satisfactorily to produce efficient general practitioners and hospital physicians and surgeons from the same mill. The qualifying curriculum and examinations, which all must pass, should be directed primarily to the production of efficient general practitioners, and much that is now crammed into the curriculum and scampered through should be relegated to postgraduate courses and examinations for higher or special diplomas.

---

### RENAL EFFICIENCY.

